# RTS,S/AS01 malaria vaccine mismatch observed among *Plasmodium falciparum* isolates from southern and central Africa and globally

**DOI:** 10.1038/s41598-018-24585-8

**Published:** 2018-04-26

**Authors:** Julia C. Pringle, Giovanna Carpi, Jacob Almagro-Garcia, Sha Joe Zhu, Tamaki Kobayashi, Modest Mulenga, Thierry Bobanga, Mike Chaponda, William J. Moss, Douglas E. Norris

**Affiliations:** 1 W. Harry Feinstone Department of Molecular Microbiology and Immunology, Johns Hopkins Malaria Research Institute, Johns Hopkins Bloomberg School of Public Health, Baltimore, MD USA; 2 Big Data Institute, Li Ka Shing Centre for Health Information and Discovery, University of Oxford, Oxford, UK; 3 Medical Research Council (MRC) Centre for Genomics and Global Health, University of Oxford, Oxford, UK; 4 The Wellcome Trust Sanger Institute, Hinxton, UK; 5 Department of Epidemiology, Johns Hopkins Malaria Research Institute, Johns Hopkins Bloomberg School of Public Health, Baltimore, MD USA; 6 Tropical Diseases Research Centre, Ndola, Zambia; 7 Université Protestante au Congo and University of Kinshasa, Kinshasa, Democratic Republic of the Congo

## Abstract

The RTS,S/AS01 malaria vaccine encompasses the central repeats and C-terminal of *Plasmodium falciparum* circumsporozoite protein (PfCSP). Although no Phase II clinical trial studies observed evidence of strain-specific immunity, recent studies show a decrease in vaccine efficacy against non-vaccine strain parasites. In light of goals to reduce malaria morbidity, anticipating the effectiveness of RTS,S/AS01 is critical to planning widespread vaccine introduction. We deep sequenced C-terminal *Pfcsp* from 77 individuals living along the international border in Luapula Province, Zambia and Haut-Katanga Province, the Democratic Republic of the Congo (DRC) and compared translated amino acid haplotypes to the 3D7 vaccine strain. Only 5.2% of the 193 PfCSP sequences from the Zambia-DRC border region matched 3D7 at all 84 amino acids. To further contextualize the genetic diversity sampled in this study with global PfCSP diversity, we analyzed an additional 3,809 *Pfcsp* sequences from the Pf3k database and constructed a haplotype network representing 15 countries from Africa and Asia. The diversity observed in our samples was similar to the diversity observed in the global haplotype network. These observations underscore the need for additional research assessing genetic diversity in *P. falciparum* and the impact of PfCSP diversity on RTS,S/AS01 efficacy.

## Introduction

Although indoor residual spraying (IRS) and insecticide treated bednets (ITNs) have dramatically decreased malaria transmission, the global impact of malaria remains high with an estimated 216 million cases reported in 2016^1,2^. Sub-Saharan Africa experiences a disproportionately high burden of *Plasmodium falciparum* malaria, even in regions with high coverage of IRS and ITNs^1,3^. Recent World Health Organization (WHO) goals aim to reduce both malaria mortality and case incidence by 90% of 2015 levels by 2030^1^. Given the inadequacy of IRS and ITNs to eliminate malaria in all transmission settings, additional tools are necessary^3^. Of particular interest is an effective vaccine which might enhance control efforts and reduce malaria associated morbidity and mortality, particularly in regions refractory to current interventions.

Circumsporozoite protein (CSP), the dominant surface protein coating infectious stage sporozoites, has been a focus of vaccine development since the observation that bites from irradiated, infectious mosquitoes induce protective immune responses^4^. *P. falciparum* CSP (PfCSP) has three distinct regions: the conserved amino (N)-terminal region, the central repeat region (CRR) comprised of 37–42 NANP repeats, and a polymorphic carboxyl (C) -terminal containing two sub-regions of high diversity known as Th2R and Th3R that elicit T-cell responses^5,6^. The CRR contains the most immunogenic B-cell sporozoite epitopes and anti-CSP antibodies induced by exposure to irradiated, live sporozoites prevent infection by binding at the CRR in animal models^7^.

Malaria vaccine development efforts have spanned multiple decades and recently culminated in the licensure of the RTS,S/AS01 vaccine by GlaxoSmithKline (GSK) in 2015. The RTS,S/AS01 vaccine is a recombinant protein vaccine containing a portion of the NANP repeats (B-cell epitopes) and the C-terminal region (B-cell and T-cell epitopes) of the PfCSP fused with hepatitis B surface antigen (HBsAg) and is administered with a novel adjuvant, AS01^8,9^. The vaccine construct is based on the *P. falciparum* 3D7 clone, which was derived from the NF54 strain isolated from a patient living near Schipol Airport in Amsterdam^8,10^. Phase III clinical trials carried out at 11 sites across seven countries in sub-Saharan Africa demonstrated an overall vaccine efficacy (estimated using negative binomial regression) against clinical malaria from month zero to study end (children: median 48 months until study end, infants: median 38 months until study end) of 36.3% in children aged 5–17 months who received 3 primary doses of RTS,S plus a booster at 20 months^11^. In 2015, the European Medicines Agent (EMA) approved the use of RTS,S/AS01^12^. The Malaria Vaccine Implementation Programme (MVIP) led by WHO will begin RTS,S/AS01 implementation in three high transmission regions of Ghana, Kenya, and Malawi in 2018 with the goals of continued evaluation of the vaccine’s impact on mortality, evaluating the feasibility of deploying the four dose vaccine series, and continued monitoring of vaccine safety^12^.

Because the gene encoding *P. falciparum* CSP (*Pfcsp*) is globally diverse^13–15^, multiple studies were conducted during the RTS,S/AS01 Phase II clinical trials to monitor *Pfcsp* haplotypes from vaccine and placebo recipients for signals of allele-specific vaccine-induced immunity. Four studies conducted in The Gambia, Kenya, and Mozambique found no evidence of allele-specific vaccine-induced immunity^16–19^. These genetic surveillance analyses relied either on Sanger sequencing^16,18^ or oligonucleotide hybridization assays to assign genotypes to *P. falciparum* isolates^17,19^. While state of the art assays at the time, the advent of affordable and scalable next generation sequencing technologies with the capacity to rapidly analyze larger sample sets has rendered both methods outdated.

Following the Phase III clinical trials, researchers used Illumina MiSeq and PacBio next generation sequencing technologies respectively to sequence both the C-terminal and CRR regions of *Pfcsp* from parasites collected from individuals vaccinated with RTS,S/AS01 or placebo at 11 Phase III trial sites^20^. Cumulative vaccine efficacy was reduced from 50.3% for parasites with a perfect *Pfcsp* C-terminal sequence match to only 33.4% for parasites with any amino acid mismatch in this region^20^. Although previous studies did not provide evidence to support the allele dependent nature of RTS,S/AS01 vaccine efficacy^16–19^, this recent analysis using technologically advanced methods and a larger sample size suggests allele-specific immunity is important in eliciting protection^20^. Further, this observation offers a potential explanation into the wide range of RTS,S/AS01 efficacies observed during Phase III clinical trials across the 11 sites (range: 22.0–74.6% against clinical malaria from month zero until the end of follow-up among children receiving three primary doses of RTS,S/AS01 plus a booster at 20 months; vaccine efficacy estimated through negative binomial regression)^11^.

In Zambia, malaria risk is heterogeneous with regions targeted for malaria elimination in the south and districts in which prevalence is greater than 50% throughout the year in the north^21^. Nchelenge District is located in northern Zambia in Luapula Province, the province with the highest malaria prevalence in children younger than five years of age (>50% by malaria rapid diagnostic test in 2014)^21^. Despite a decade of malaria control interventions in Nchelenge District, including implementation of ITNs and IRS, malaria transmission remains holoendemic, with prevalence greater than 50% through 2017 (unpublished data)^3,22^. While Zambia scales up to meet its 2021 malaria elimination goal, it is important to consider the utility of introducing RTS,S/AS01 into the current arsenal of tools to reduce malaria morbidity and mortality in this region. Towards this goal, the genetic diversity of the C-terminal *Pfcsp* was characterized with respect to the vaccine strain in parasites collected from the border of northern Zambia and the Democratic Republic of the Congo (DRC). Further, the genetic diversity of the C-terminal *Pfcsp* sequences in this study was contextualized within global *Pfcsp* diversity from the Pf3k database.

## Methods

### Sample Collection, DNA Extraction, and Quantification

Dried blood spot (DBS) samples were collected at one time point from consenting individuals living in randomly selected households during June and July 2016 in Nchelenge District, Zambia as well as two villages, Kilwa and Kashobwe, located directly across the Zambian border in the DRC. One hundred and three DBS samples from unique individuals (64 Zambian participants and 39 DRC participants) ranging in age from 8 months to 72 years (mean 17.7 years) and identified to be *P. falciparum* positive through qPCR screening of the DBS in Zambia were shipped to the Johns Hopkins Bloomberg School of Public Health, extracted using 20% Chelex, and quantified using *P. falciparum lactose dehydrogenase* (*Pfldh*) qPCR^23^.

### Amplicon Generation and Sequencing

A 300-bp amplicon containing the C-terminal *Pfcsp* (839-1,139-bp, clone 3D7 0304600.1, PlasmoDB^24^) was amplified from *P. falciparum* positive samples using the forward primer GACAAGGTCACAATATGCCAAA and reverse primer ACATTAAACACACTGGAACATTTTTC fused with Illumina MiSeq adapter sequences for library indexing during PCR^25^. PCR amplification reaction components included: 10 μL DNA template, 12.5 μL KAPA Hifi HotStart ReadyMix (Kapa Biosystems, Wilmington, Massachusetts), 0.25 μL each of 20 μM forward and reverse primers containing Illumina adapters, and 2 μL of 25 μM magnesium chloride. PCR cycling conditions were 95 °C for 5 minutes followed by 30 cycles of 98 °C for 20 seconds, 61 °C for 30 seconds, 72 °C for 1 minute, 72 °C for 5 minutes, and a holding step at 4 °C. Amplicon size (300-bp) was verified using TapeStation (Agilent 4200, Santa Clara). Seventy-five percent of the 103 samples successfully generated 300-bp *Pfcsp* amplicons. Among samples with >50 p/μL by qPCR, the amplicon generation success rate was 100%; for samples with <50 p/μL by qPCR, the success rate was 7%. The arithmetic mean of parasite copy number for samples that failed to generate amplicons was 11.1 p/μL (range: 0.5 p/μL–44.5 p/μL) compared with arithmetic mean 8,666 p/μL (range: 4.0 p/μL–81,196.4 p/μL) for samples for which amplicon generation was successful.

Amplicons were uniquely barcoded in a subsequent PCR reaction containing Nextera (Illumina, San Diego, California) indexes as described by Illumina^25^. Indexed amplicon sizes were verified using TapeStation, purified using AMPure beads (Beckman Coulter, Brea, California), and quantified using PicoGreen (ThermoFisher Scientific, Waltham, Massachusetts). The *Pfcsp* amplicons were normalized and combined into a single pool for 300-bp paired end sequencing on a MiSeq at the Sequencing and Synthesis Facility at the Johns Hopkins School of Medicine.

### Bioinformatic Processing and Analysis

Forward and reverse reads were merged using FLASH^26^, trimmed for quality (sliding window = 50-bp, step size = 5-bp, quality threshold = 20) and collapsed by haplotype using SeekDeep’s^27^ default Illumina settings and allowing for one high quality mismatch within individuals. Samples included in the final analysis were supported by high read coverage, with an average of 29,439 reads. Haplotypes found to represent at least 1% of a sample were considered in the final analysis in order to minimize the inclusion of false positive haplotypes. The number of genetically distinct parasite haplotypes per individual, or complexity of infection (COI), was determined by the number of unique haplotype clusters per individual, as estimated by SeekDeep. The *Pfcsp* sequences were translated to amino acid sequences and aligned to the 3D7 vaccine reference strain (3D7 0304600.1, PlasmoDB)^24^ in Geneious (version 9.1.5). The number of amino acid differences was calculated for each sequence and the 3D7 reference sequence for the 84 amino acids in the C-terminal amplicon (amino acids 288–371).

### Data Availability Statement

The unique DNA sequences obtained from this study have been deposited in GenBank (accession numbers MG715504-MG715555).

### Pf3k Sequence Acquisition and Global Diversity Analysis

Global *Pfcsp* diversity was examined by mining the MalariaGEN Pf3k Project (release 5)^28^ which includes 2,512 *P. falciparum* full genomes from 14 countries worldwide. We retrieved all genetic variants on chromosome 3 available in Pf3k in variant call format (VCF) from release 5.1. Variant calls were made using GATK best practice haplotypeCaller^29,30^. Variants in the C-terminal *Pfcsp* region (nucleotides 866–1,113) were extracted for the 1,147 monoclonal infections from Africa and Asia. The individual *Pfcsp* haplotype sequences for the 1,365 multi-clonal samples were reconstructed using DEploid^31^ with appropriate reference panels of mono-clonal samples from the Pf3k dataset^29^. We constructed a network based on the method by Templeton, Crandall, and Sing (TCS) using PopArt^32^ to assess genealogical relationships between the global *Pfcsp* haplotypes found in Pf3k and the haplotypes from Zambia and the DRC^33,34^. Genetic diversity metrics were calculated using DnaSP (version 6.10.01). We compared sequences between African and Asian countries in terms of diversity and for evidence of population differentiation by calculating *F*_ST_. Similarly, we compared samples from east and west African countries and calculated *F*_ST_ for signatures of population structure. For the purposes of comparing east and west Africa, we grouped samples in the DRC with east African samples.

### Ethics Approval and Informed Consent

This research was approved by the Institutional Review Board at the Johns Hopkins Bloomberg School of Public Health in Baltimore, Maryland, USA, the Ethics Review Committee for the Tropical Diseases Research Centre in Ndola, Zambia, and by Le Comité d'éthique de l’Université Protestante au Congo in Kinshasa, the Democratic Republic of the Congo. All studies were conducted in accordance with the ethical guidelines set forth by the aforementioned review boards. All adults who participated in these studies gave informed consent. All child participants gave assent and had parental consent for participation.

## Results

**Figure 1:**
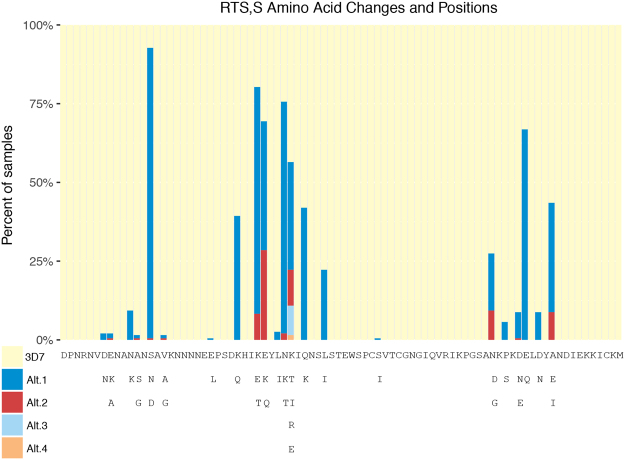
RTS,S Amino Acid Changes and Positions. The 84 amino acids (positions 288–371) comprising the C-terminal amplicon are represented by columns in the bar-chart. The percentage of samples sharing the 3D7 amino acid are represented in pale yellow. Non-3D7 amino acid alternatives are represented in descending order of frequency in dark blue, red, light blue, or orange. Below the bar-chart, the 3D7 amino acid sequence is shown, with positions corresponding to the coordinates above. The substitutions at each of the 84 positions are enumerated below the 3D7 sequence.

Of the 103 DBS samples extracted from our collections in Zambia and the DRC, 77 yielded suitable *Pfcsp* amplicons for sequencing. Fifty five of the 77 samples sequenced passed quality filtering steps implemented by FLASH and SeekDeep. Two samples were excluded from the analysis for lacking full length *Pfcsp* sequences. Overall, 193 PfCSP haplotype sequences from 53 individuals were characterized, corresponding to 52 unique haplotypes (Table 1) ranging in population frequency from 1 to 22 (mean 3.7) observations across the 53 individuals. The 53 individuals were infected with a mean of 3.6 genetically distinct parasite haplotypes (range: 1–10). Of the 193 parasite sequences characterized from the 53 human samples, only ten matched the 3D7 haplotype at all 84 amino acids (5.2%). The median number of amino acid differences across all 193 parasite haplotypes in comparison to 3D7 was seven. Of the 10 3D7-type parasites, eight were found in individuals from Zambia and two were found in individuals from the DRC. The proportion of individuals harboring a 3D7-type parasite were similar between countries (Zambia: 25.8%, DRC: 9.1%, Pearson’s chi-squared test of equal proportions: p = 0.24) and between sampling sites (Kilwa: 0%, Kashobwe: 13.3%, Nchelenge lakeside: 31.8% Nchelenge inland: 11.1%, Pearson’s chi-squared test of equal proportions: p = 0.20). The frequency of 3D7 (vaccine) matched parasites was lower than previously reported in other African countries^13,17^. Furthermore, all 10 3D7-type parasites were found in the context of polyclonal infections (range 3–7 haplotypes). The 3D7-type was not the major haplotype in nine of these ten (90%) polyclonal infections (median relative abundance of 3D7-type haplotypes in an individual = 5%; range = 1–24%). Divergent regions of the PfCSP amplicon from 3D7 were visualized by plotting the percentage of samples sharing the 3D7 reference amino acid at each of the 84 amino acid positions (Fig. 1). Twenty-two polymorphic amino acid positions were identified, eight of which were within the Th2R region (amino acids 311–327) and six were contained in Th3R (amino acids 352–363).

**Table 1:** Amino Acid Haplotype Frequencies.

Rank order of unique haplotypes	Frequency	Number of AA Differences
1	22	8
2	15	5
3	13	8
4	11	7
5	10	0
6	10	6
7	8	7
8	7	7
9	7	8
10	7	8
11	5	7
12–15	4	5–7
16–21	3	4–8
22–33	2	2–9
34–52	1	5–10

Summarizes the frequency distribution of the observed 52 unique C-terminal haplotypes from Zambia and the DRC. Haplotypes are listed in descending order of frequency, with Rank 1 representing the most common haplotype. The number of amino acid (AA) differences was calculated against 3D7 reference 0304600.1 (PlasmoDB).

The *Pfcsp* nucleotide diversity observed within Nchelenge District, Zambia, and Kilwa and Kashobwe in the DRC appeared to be representative of African *Pfcsp* in the TCS haplotype network constructed using samples from this study in addition to 3,809 sequences from the Pf3k database. In total, we identified 393 unique *Pfcsp* haplotypes, of which seven account for 51.3% of all the 4,002 sequences analyzed in this study.

**Figure 2:**
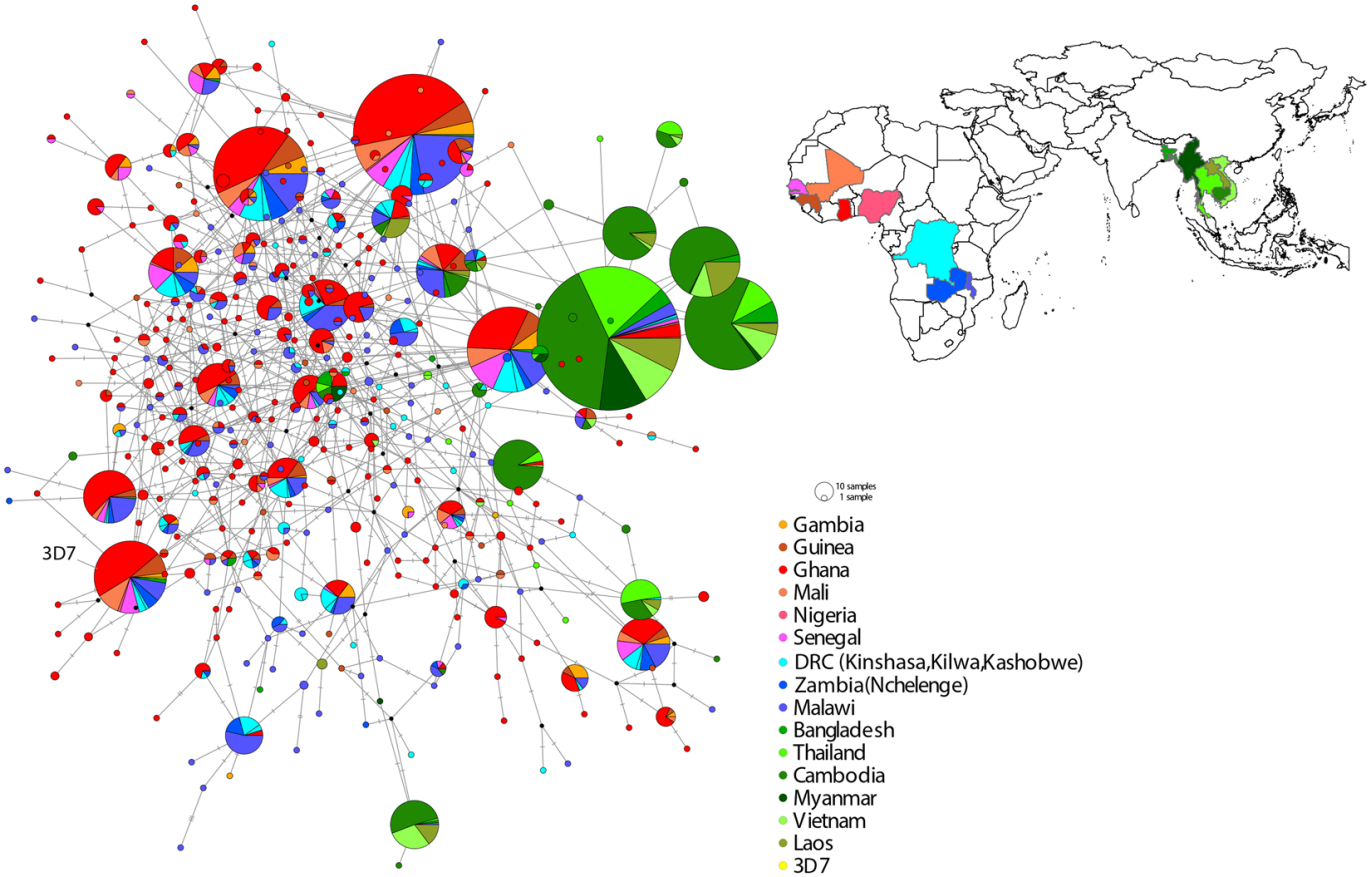
African and Asian *Pfcsp* Haplotype Network. Templeton, Crandall, and Sing (TCS) network summarizing the global diversity of the C-terminal *Pfcsp* from 4,002 sequences. Circles represent unique nucleotide haplotypes, and circles are scaled according to the frequency which the haplotype was observed. Vaccine strain 3D7 (0304600.1, PlasmoDB) is included for reference. Haplotype colors match the geographic origin of the samples depicted on the map.

No clear population structure was identified between east and west African isolates (F_ST_ = 0.008), although signatures of moderate population differentiation were observed between African and Asian samples (Fig. 2), with F_ST_ = 0.163. We observed higher nucleotide diversity among African isolates than in Asian isolates, but no difference between east and west African isolates (Table 2). Among all African isolates analyzed, the 3D7 haplotype represented only 5.3% of the African 2,635 sequences. Including both Asian and African isolates, the 3D7 haplotype represented only 3.6% of 4,002 sequences. Among the Asian isolates, only three sequences matched the 3D7 haplotype (0.2%), all reported from Bangladesh.

**Table 2:** *Pfcsp* Global Diversity Statistics.

Continent	Region	Country	n	h	S	K	Hd	π
Africa			2,635	370	33	4.11	0.948	0.117
	East Africa		910	181	26	4.05	0.951	0.116
		DRC	268	64	22	4.01	0.950	0.114
		Malawi	534	150	25	3.99	0.951	0.114
		Zambia	108	31	19	4.45	0.933	0.127
	West Africa		1725	264	31	4.11	0.945	0.117
		The Gambia	105	23	20	4.19	0.905	0.120
		Ghana	1109	232	29	4.17	0.948	0.119
		Guinea	175	49	21	3.94	0.939	0.112
		Mali	166	53	24	3.84	0.935	0.110
		Nigeria	7	4	6	2.95	0.857	0.084
		Senegal	163	35	22	4.04	0.928	0.116
Asia			1,367	44	20	2.58	0.780	0.074
		Bangladesh	80	20	15	2.54	0.859	0.072
		Cambodia	752	23	17	2.66	0.807	0.076
		Laos	129	11	13	2.68	0.799	0.077
		Myanmar	81	6	9	0.65	0.349	0.019
		Thailand	197	14	12	2.09	0.589	0.060
		Vietnam	128	15	15	3.07	0.759	0.088

Summarizes the samples included in the *Pfcsp* network analysis, including 193 samples sequenced in this study and 3,809 from Pf3k database. n = number of sequences, h = number of unique haplotypes, S = number of segregating sites (out of total possible 35), K = average number of pairwise nucleotide differences, Hd = haplotype diversity, π = nucleotide diversity.

## Discussion

RTS,S vaccine efficacy has been shown to decline from 50.3% for vaccine matched parasite CSP haplotypes to 33.4% for unmatched haplotypes^20^. In our study sites, only 10 parasite sequences of the 193 recovered (5.2%) were an exact match to the amino acid sequence in the C-terminal CSP region of the vaccine strain. The proportion of individuals harboring 3D7-type parasites was similar between Zambia and the DRC. The frequency of vaccine matched parasites observed in this study is lower than previously reported in other African countries^13,17^.

It was previously demonstrated that RTS,S vaccine efficacy declined substantially for parasites not matching 3D7 in the C-terminal region of *Pfcsp*^20^, and that vaccine efficacy declines as the number of amino acid differences increases^20^. Therefore, the implication of parasites along the Zambia-DRC border differing from 3D7 at a median of seven amino acids is potentially significant. All 10 3D7-type parasites identified in our study occurred in the context of polyclonal infections (range 3–7 haplotypes). While Neafsey *et al*. evaluated the proportion of infections containing a 3D7 matched parasite haplotype as a function of COI^20^, how vaccine efficacy differs between monoclonal 3D7-type infections, infections where 3D7 is the major of multiple haplotypes, and infections where 3D7 is the minor of multiple haplotypes has not yet been studied. Additional studies aimed at clarifying the effect of polyclonal infections on vaccine efficacy are warranted.

RTS,S/AS01, the only currently licensed malaria vaccine, is based on the sequence of just one parasite clone, 3D7, of African origin^35^. Given that the vaccine is based on an African parasite clone, considering to what extent circulating parasites from Asia differ relative to those in Africa can provide us with insight into how well RTS,S/AS01 may perform if implemented in Asian countries. In this study, we observe moderate population differentiation between Asian and African isolates (F_ST_ = 0.163). Previous studies that aimed to assess global *Pfcsp* diversity identified population structure between isolates from Africa and Asia, consistent with our observations^14,15^. Barry *et al*. used *Pfcsp* sequences from GenBank (n = 604) and characterized global diversity in an approximately similar C-terminal *csp* region (nucleotides 909–1140) to this study (nucleotides 866–1113)^15^. Although the *Pfcsp* network created by Barry *et al*. included only five of the sixteen countries represented in this study, the overall pattern of high global *Pfcsp* diversity was consistent across the two studies, strengthening the conclusions presented in both analyses. Notably, previous analyses of global *Pfcsp* diversity have not included isolates from multiple east African countries^14,15,36^. Here, we include 910 *Pfcsp* sequences from three east African countries, providing, to our knowledge, the first large scale characterization of *P. falciparum* genetic diversity in relation to the RTS,S/AS01 vaccine across multiple countries in this historically understudied area. Further, previous research has focused on describing *Pfcsp* diversity from monoclonal malaria infections^13^ which may underestimate true population genetic diversity. This study characterizes *Pfcsp* haplotypes from multiple clones present in polyclonal infections, providing a more complete analysis of population diversity.

Ideally, an effective malaria vaccine would provide protection against the majority of circulating parasites across multiple geographic regions. Our data provide evidence that *Pfcsp* exhibits high genetic diversity both locally and globally. Interestingly, the prevalence of the 3D7-type parasite strains in our study area (5.2%) is the same as that across all of the African countries included in the Pf3k dataset (5.3%, n = 140/2635). Among Asian isolates, the 3D7 haplotype is even less frequently observed (n = 3/1367) at only 0.2% prevalence. In fact, Bangladesh is the only Asian country in the Pf3k dataset in which the 3D7 haplotype was observed. These data support previous observations that C-terminal *Pfcsp* is diverse globally, and that 3D7-type parasites are more frequently found in African countries than Asian counties^14,15^. The high degree of global genetic *Pfcsp* diversity may potentially reduce RTS,S/AS01 vaccine effectiveness, particularly in Asian countries where 3D7 was not or only rarely observed. Monitoring differential vaccine efficacy by PfCSP haplotype during possible future RTS,S/AS01 implementation programs will be valuable.

Publically available sequence databases provide unparalleled opportunities to understand global pathogen population genetics. However, it is important to acknowledge the limitations of drawing inferences from non-randomly sampled sequences. Notably, countries, as well as regions within countries, have unequal rates of sample deposition into databases, leading to a geographically biased set of sequences which over-represent genotypes from a small number of geographic foci while under-representing large swaths of the globe. Further, the conclusions drawn from sequences obtained from any given sequence repository are subject to change as sample sizes and geographic distributions are continually updated and expanded. Finally, the sequences obtained from Pf3k represent a multitude of sampling strategies, time periods, and sequencing technologies, which prevent samples from various regions from being optimally comparable. These samples come from patients across a spectrum of ages rather than specifically from children who are the recipients of the RTS,S/AS01 vaccine. However, despite these inherent limitations, sequence databases are powerful tools capable of elucidating global patterns in pathogen population genetic diversity. These resources, coupled with functional laboratory studies as well as observational field research, have a critical role to play in vaccine development efforts against a repertoire of global pathogens, including malaria. In the context of this study and with these limitations, we recognize that we have likely underestimated the true global diversity of *Pfcsp*.

The data presented here highlight the diversity of C-terminal *Pfcsp*, particularly in sub-Saharan Africa which is a key target region for malaria vaccination programs. This study underscores the importance of incorporating population genetic studies into future malaria vaccine design, laboratory and clinical evaluation. Most importantly, assessing the diversity of C-terminal *Pfcsp* should be a component of the RTS,S/AS01 Malaria Vaccine Implementation Programme and in further refining our understanding of how genetic diversity affects RTS,S/AS01 efficacy.
